# MAPkinases regulate secondary metabolism, sexual development and light dependent cellulase regulation in *Trichoderma reesei*

**DOI:** 10.1038/s41598-023-28938-w

**Published:** 2023-02-02

**Authors:** Miriam Schalamun, Sabrina Beier, Wolfgang Hinterdobler, Nicole Wanko, Johann Schinnerl, Lothar Brecker, Dorothea Elisa Engl, Monika Schmoll

**Affiliations:** 1 Center for Health and Bioresources, Bioresources Unit, AIT Austrian Institute of Technology GmbH, Konrad Lorenz Strasse 24, 3430 Tulln, Austria; 2 MyPilz GmbH, Wienerbergstrasse 55/13-15, 1120 Vienna, Austria; 3 Department of Botany and Biodiversity Research, University of Vienna, Rennweg 14, 1030 Vienna, Austria; 4 Department of Organic Chemistry, University of Vienna, Währinger Strasse 38, 1090 Vienna, Austria; 5 Division of Terrestrial Ecosystem Research, Department of Microbiology and Ecosystem Science, University of Vienna, Djerassiplatz 1, 1030 Vienna, Austria

**Keywords:** Fungal genetics, Fungal physiology

## Abstract

The filamentous fungus *Trichoderma reesei* is a prolific producer of plant cell wall degrading enzymes, which are regulated in response to diverse environmental signals for optimal adaptation, but also produces a wide array of secondary metabolites. Available carbon source and light are the strongest cues currently known to impact secreted enzyme levels and an interplay with regulation of secondary metabolism became increasingly obvious in recent years. While cellulase regulation is already known to be modulated by different mitogen activated protein kinase (MAPK) pathways, the relevance of the light signal, which is transmitted by this pathway in other fungi as well, is still unknown in *T. reesei* as are interconnections to secondary metabolism and chemical communication under mating conditions. Here we show that MAPkinases differentially influence cellulase regulation in light and darkness and that the Hog1 homologue TMK3, but not TMK1 or TMK2 are required for the chemotropic response to glucose in *T. reesei*. Additionally, MAPkinases regulate production of specific secondary metabolites including trichodimerol and bisorbibutenolid, a bioactive compound with cytostatic effect on cancer cells and deterrent effect on larvae, under conditions facilitating mating, which reflects a defect in chemical communication. Strains lacking either of the MAPkinases become female sterile, indicating the conservation of the role of MAPkinases in sexual fertility also in *T. reesei*. In summary, our findings substantiate the previously detected interconnection of cellulase regulation with regulation of secondary metabolism as well as the involvement of MAPkinases in light dependent gene regulation of cellulase and secondary metabolite genes in fungi.

## Introduction

To survive in a competitive habitat, organisms evolved complex signaling pathways to properly react to a changing environment while optimally balancing resources for survival and growth. Especially sunlight profoundly impacts organsims living on earth and if light perception or–response machineries are impaired, severe consequences for fitness or even survival were observed^1,2^. The conserved mitogen activated protein (MAP) kinase pathways play a central role in signal transmission and–integration in eukaryotes from fungi to mammals^3,4^.

MAPkinase cascades have been subject to intense research efforts in eukaryotes, which revealed their contribution to virtually all crucial physiological processes from growth, response to hyphal injury, reproduction, stress response, secondary metabolite production to metabolism and light response^4–8^.

In filamentous fungi, three major MAPkinase pathways are known: The pheromone response pathway^9^, the cell wall integrity pathway^10^ and the osmoregulation pathway^11^. MAPkinase pathways each consist of three protein kinases, a MAPkinase, a MAPkinase kinase (MAPKK) and a MAPkinase kinase kinase (MAPKKK) which form a phosphorylation cascade^12,13^. This 3-tiered modular construct is likely positively selected during evolution^14^. Stepwise phosphorylation enables signal integration at every stage and is required for activation. Subcellular localization of MAPkinases is crucial for their function and establishment of regulatory feedback loops^15^. Thereby, MAPkinases are known to be subject to feedback inhibition, which contributes to signal fidelity and is often achieved by phosphatases dephosphorylating and hence inactivating MAPkinases^13^.

Evaluation of the functions of the pheromone MAPkinase pathway in *Aspergillus flavus* showed that its members (*steC, mkkB, mpkB* and *steD*) act as a complex and are required for aflatoxin B1 production, while in the respective deletion mutants an increase in production of leporin B and aspergillicins was observed^9^. Mechanistic investigation of the role of this pathway in aflatoxin production revealed that the regulatory impact of this kinase targeted biosynthesis of precursors rather than regulation of the aflatoxin gene cluster^16^. In contrast, deletion of the Hog1-type MAPkinase SakA in *A. flavus* caused an increase in aflatoxin production^17^. Components of the cell wall integrity pathway are involved in regulation of secondary metabolism in many fungi, where they are often required for their production^10^. Already these few examples show that regulation of secondary metabolism is a common trait for the function of MAPkinase pathways in fungi.

Fungi use chemicals to communicate with mating partners and competitors^18,19^. Importantly, a considerable part of the functions of MAPKs is aimed at appropriate communication with the environment, which is crucial not only for competition, but also for virulence and pathogenicity^7,20^. While the correct function of such a communication can be detected relatively easily by genetic screenings and microscopic analysis, the compounds responsible for this interaction—the chemical(s) eliciting the response—are much harder to identify. One example is the chemotropic growth of the phytopathogen *Fusarium oxysporum* towards plants which is regulated by the CWI MAPkinase pathway, for which a peroxidase was found to be responsible^21,22^, which is however unlikely to be the chemical that is detected. Another case of chemical communication is represented by the rhythmic activation of MAPkinases upon fungal communication between *Neurospora crassa* hyphae^23^. This interaction mechanism is conserved between *N. crassa* and *B. cinerea*^24^ although also here the chemical compounds mediating this interaction are not yet known.

The rotation of earth causing night and day represents one of the most important environmental cues for life, including fungi^1^. Thereby, organisms do not simply respond the the increasing light intensity in the morning, but they prepare for both dusk and dawn using a circadian clock, which keeps running even in the dark^25,26^. Light is essential for entraining the clock and a light pulse resets the clock, which impacts the whole gene regulation machinery as well^25,27^. MAPkinases play an important role in circadian rhythmicity due to their rhythmic activation and their role in phosphorylation of clock proteins^28^. They are a crucial output pathway of the circadian clock^29^.

Both upon constant light conditions as well as during a time course reflecting circadian rhythmicity, discrepancies between mRNA abundance and protein abundance were observed^30,31^ and also metabolism related gene oscillate during the circadian day^32^. With respect to circadian rhythmicity, it is particularly interesting, that the rhythmic activation of the osmosensing MAPK pathway influences regulation of translation in dependence of osmotic stress^33^.

The Hog-pathway transmits the phytochrome-related red light signal independently of its function as a stress signaling factor in *Aspergillus nidulans*^34^.

The genus *Trichoderma* comprises a diverse array of mostly benefical fungi, which comprise plant symbionts and industrial workhorses for enzyme production^35–40^.

In *Trichoderma*, light profoundly influences physiology^41,42^ with respect to growth^43–45^, asexual and sexual development^46,47^, regulation of plant cell wall degrading enzymes^48^, secondary metabolism^49,50^ and stress response^51–53^. Moreover, the MAPkinase encoding gene *tmk3* is induced by light in a photoreceptor dependent manner in *T. atroviride*^54^ and in *T. reesei*^55^ and early, transient phosphorylation of TMK3 occurs in *T. atroviride*^56^. Also the photoreceptor gene *env1* and the photolyase gene *phr1* have strongly increased transcript levels in a strain lacking *tmk3*, hence indicating a dampening effect of the HOG pathway on light response and potentially increased light sensitivity in deletion strains^56^.

In *S. cerevisiae*, the MAPkinase of the pheromone pathway is Fus3^12^, the homologue of *T. reesei* TMK1. Upstream of the *S. cerevisiae* MAPkinase cascade, the G-protein beta and gamma subunit mediate transmission of the pheromone signal to the MAPkinases^5^. In filamentous fungi not only Fus3 homologues, but also components of other MAPkinase pathways were shown to be required for proper sexual development. The MAPkinase mediating the cell wall integrity (CWI) pathway in *N. crassa* was found to be required for formation of protoperithecia if a strain was meant to assume the female role in a cross^57^. Moreover, Slt2 homologues are required for female fertility in *F. graminearum*^58^ and *Magnaporthe grisea*^59^. In *F. graminearum*, lack of of the Hog-pathway MAPkinase blocked sexual development^60^. Crosstalk was observed among the CWI and pheromone response pathways in *N. crassa*^61^. Hence, while the pheromone response pathway has a central function in sexual development, all three MAPkinases contribute to the process of sexual reproduction.

Induction of sexual development in *T. reesei* deviates from methods in other fungi in that so far, no protoperithecia or similar early female stages were observed in this fungus^62,63^. However, due to the inability of the prominent wild-type strain QM6a to assume the female role in a cross, which is due to a defect in the scaffolding protein HAM5^64,65^, is considered female sterile^67^.

In *Trichoderma*, three MAPkinase pathways were detected, which are conserved in the genus^40,67^. Early investigations showed that *T. virens* TmkA and TmkB are required for full antagonistic potential against fungal phytopathogens^68,69^ and TmkA is needed for inducing full systemic resistance^70^. In *T. atroviride*, lack of Tmk1 reduced mycoparasitic activity, yet higher antifungal activity attributed to low molecular weight substances including 6-pentyl-α-pyrone (6PP) and peptaibol antibiotics^71^. Recently, *T. atroviride* Tmk3 and Tmk1 were implicated in polarity stress response during hyphal interaction upon mycoparasitism and the chemotropic interaction between individual hyphae in this process^72^. Another case of antagonism was shown for *T. atroviride* with *Drosophila melanogaster* larvae, which fed on the fungal mycelium. Tmk3 was required for secondary metabolite production in *T. atroviride*, which was the reason for larvae preferentially feeding on a *tmk3* mutant, although the mortality of larvae doing so was increased compared to feeding on the wild-type^73^. Furthermore, *T. atroviride* Tmk3 was required for proper response to cell wall stress, especially upon exposure to light^56^, which suggests a certain interrelationship of the cell wall integrity pathway (represented by Tmk2) and the osmosensing pathway.

Investigation of the functions of the MAPkinase pathways in *T. reesei* as well as selected upstream signaling processes revealed roles in cell wall integrity, stress response, glycogen accumulation and asexual development^74–77^. Previously, TMK1 (Fus3-like), TMK2 (Slt2-like) and TMK3 (Hog1-like) were shown to impact regulation of cellulase gene expression: TMK3 was reported to exert a strongly positive influence on cellulase production^76^, while the influence of TMK2 on transcript abundance of cellulase genes is minor, despite its negative influence on secreted cellulase activity^74^. TMK1 also negatively influences cellulase production^75,77^, although a positive effect of TMK1 was shown on transcript levels of major cellulase and xylanase genes^77^.

Despite the fact that the influence of light on MAPKinase dependent regulation of stress response and secondary metabolism was shown previously, this environmental cue was not considered in previous studies of the topic with *T. reesei*. Consequently, we investigated the impact of light on regulation of cellulase production and we show significant differences between growth in light and growth in darkness. Our study further revealed that MAPkinases are required for female fertility upon mating in *T. reesei* and that MAPkinases differentially impact secondary metabolite production under mating conditions, hence reflecting an influence on chemical communication.

## Results

Information on environmental cues is transmitted via multiple signaling cascades in fungi, one of which are the MAPkinase cascades. Although the MAPkinase genes of *T. reesei* do not show significant regulation by light^49,78^, previous work revealed an involvement of phosphorylations in general and specifically also by MAPkinase cascades in light response and circadian rhythmicity^25,28^. Additionally, we showed that the random mutant QM9414 is less light sensitive with respect to cellulase production than the wild-type strain QM6a^79^. Therefore we deleted the MAPkinase encoding genes *tmk1*, *tmk2* and *tmk3* in the wild-type background of QM6a by replacement with the hygromycin selection marker cassette^80^. Througout our study, we investigated the phase of active growth and cellulase production of QM6a, which grows somewhat more slowly than QM9414 and produces lower levels of cellulases, but has the advantage that the machinery of cellulase regulation associated signaling and gene regulation is not altered.

### MAPkinases impact growth and sporulation

**Figure 1:**
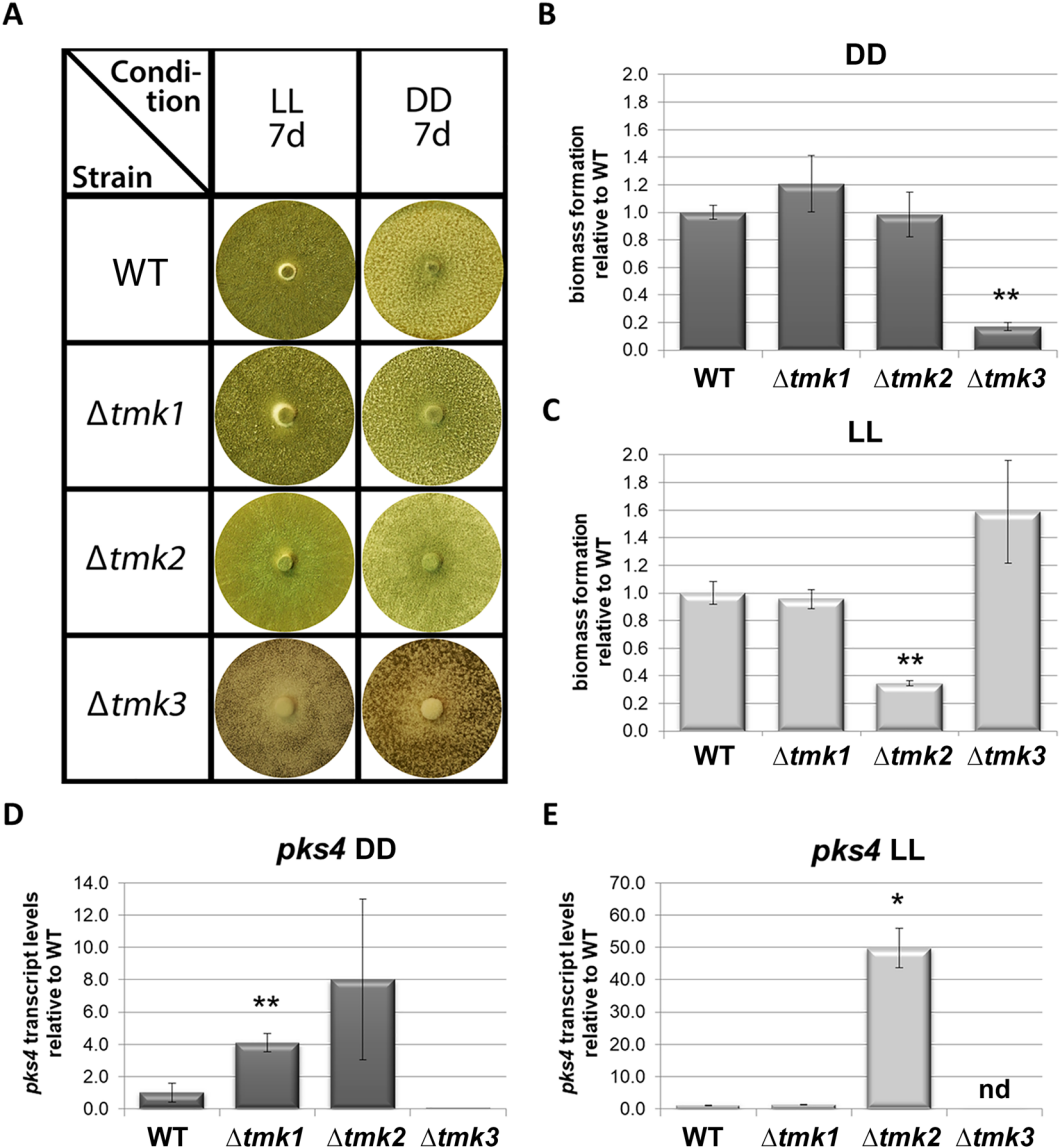
Relevance of MAPkinases for phenotype and biomass formation. (**A**) MAPkinase mutant strains on MEX agar plates in constant light (LL) and constant darkness (DD) after 7 days at 28 °C. (**B**,**C**) Biomass formation relative to wild-type (WT) QM6aΔ*ku80* upon growth on 1% cellulose in (**B**) constant darkness and (**C**) constant light. (**D**,**E**) Transcript levels of the polyketide synthases gene *pks4* upon growth on 1% cellulose in (**D**) constant darkness and (**E**) constant light.

As expected, *tmk1*, *tmk2* and *tmk3* were not essential in QM6a and grew well on malt extract agar plates (Fig. 1A). Analysis of biomass formation in liquid cultivations with cellulose as carbon source revealed strikingly different impacts in constant light and constant darkness. While in darkness Δ*tmk3* formed considerably less biomass (Fig. 1B), a similar effect was observed in light for Δ*tmk2* (Fig. 1C). This clear difference in the functions of TMK2 and TMK3 in modulating growth in light and darkness strengthens the need for cultivation under controlled light conditions. Moreover, the three MAPkinase pathways of *T. reesei* obviously exert signal transmission tasks for which it is crucial whether they grow in the dark or in light.

We also found that lack of *tmk3* in the genome causes abolishment of the typical green pigmentation of spores (Fig. 1A), which is in agreement with data from *T. atroviride*^56^. Hence, we were interested whether this is due to an impact of MAPkinases on regulation of *pks4*, the polyketide synthase responsible for this pigmentation^81^.

RTqPCR confirmed our hypothesis (Fig. 1D,E), showing that deletion of *tmk3*, which results in a white phenotype, also correlates with abolishment of *pks4* transcription in light and darkness. Interestingly, we also found that *pks4* transcript levels are strongly increased in a strain lacking *tmk2*, both in light and darkness and that Δ*tmk1* also shows elevated *pks4* levels only in darkness. Consequently, MAPkinases crucially impact spore pigmentation, both in light, as the preferred sporulation condition and in darkness.

### TMK3 is required for chemotropic response to glucose

Glucose represents an important nutrient for *T. reesei*, which represses cellulase gene expression and elicits carbon catabolite repression^82,83^. However, genome analysis revealed that *T. reesei* lacks a direct homologue of the prototypical glucose sensors GPR-4 or Git1^67^. Investigation of G-protein coupled receptors (GPCRs) implicated two class XIII (DUF300 domain) GPCRs, CSG1 and CSG2 in glucose sensing due to their impact on cellulase regulation on cellulose and lactose^78^. This function was supported by the requirement of CSG1 and CSG2 for chemotropic responses to specific concentrations of glucose^84^. Since a role in chemotropic reaction to glucose was shown for FMK1, the *Fusarium oxysporum* homologue of filamentation pathway MAPkinase^22^, we were interested in the role of *T. reesei* MAPkinases in chemotropic reactions to glucose.

**Figure 2:**
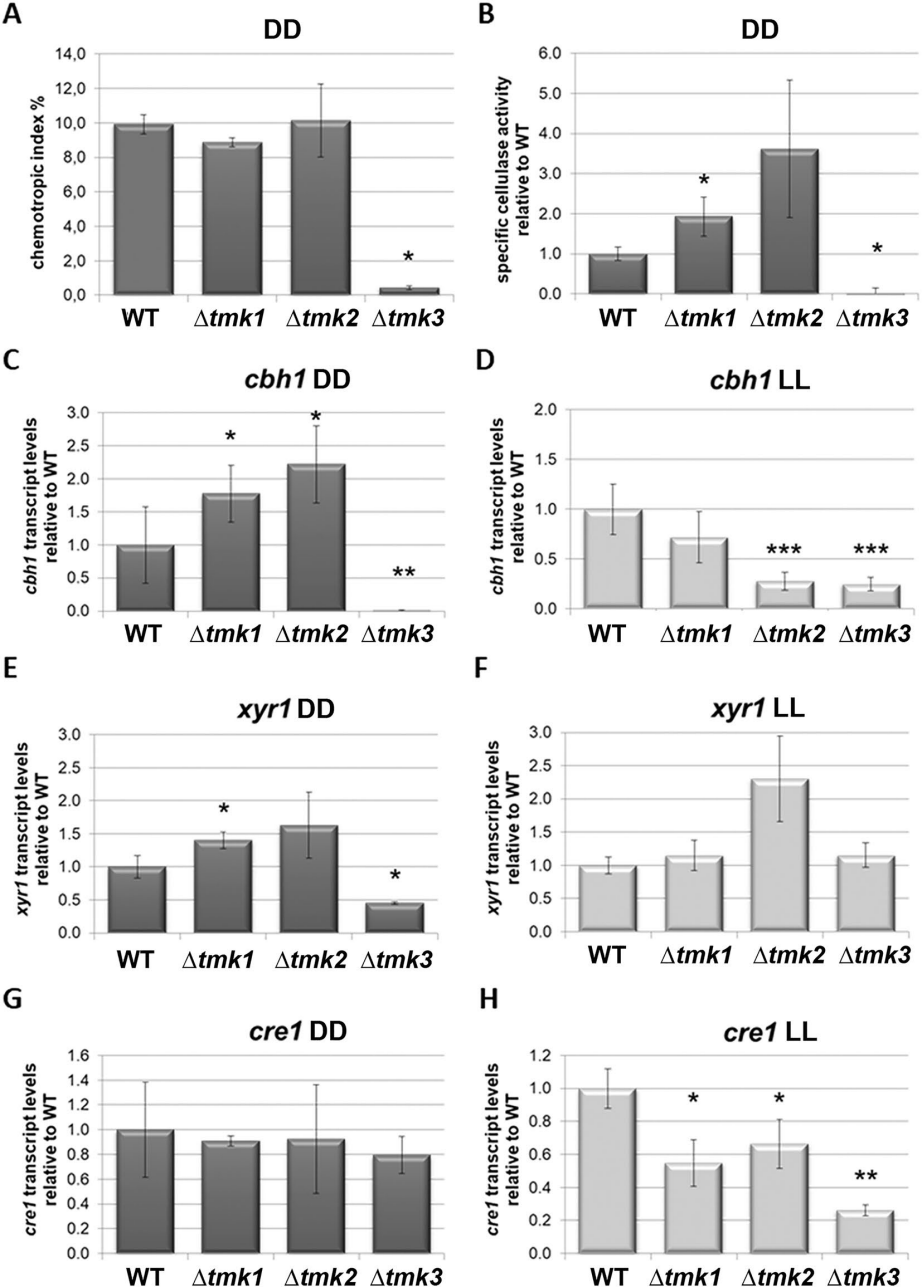
Relevance of MAPkinases for chemotropic response and cellulase regulation. (**A**) Chemotropic response of MAPkinase mutant strains to 1% glucose. (**B**) Specific cellulase activity upon growth on 1% cellulose in darkness. (**C**,**D**) Transcript levels of *cbh1* upon growth on 1% cellulose (**C**) in constant darkness and (**D**) constant light. (**E**,**F**) Transcript levels of *xyr1* upon growth on 1% cellulose (**E**) in constant darkness and (**F**) constant light. (**G**,**H**) Transcript levels of *cre1* upon growth on 1% cellulose (**G**) in constant darkness and (**H**) constant light.

Interestingly, in *T. reesei* TMK3, but not TMK1, the homologue of FMK1, is required for chemotropic response to glucose. As for the *F. oxysporum* homologue MPK1^22^, lack of the cell wall integrity pathway MAPkinase TMK2 in *T. reesei* does not perturb chemotropic response to glucose (Fig. 2A).

Since also the GPCRs CSG1 and CSG2 are required for chemotropic reactions to glucose^84^, the signaling pathway triggering this reaction in *T. reesei* might not be exclusively channeled through the G-protein pathway but may be subject to biased signaling^85^.

### MAPkinases regulate cellulase transcription and secreted activity differentially in light and darkness

An involvement *of T. reesei* MAPkinases in cellulase regulation was shown previously^74–76^. However, in these studies, the relevance of light for cellulase regulation was not considered and *T. reesei* TU-6, a parental strain derived from QM9414, with decreased and probably altered light response^79^ was used. Therefore, we aimed to evaluate these previous results under controlled light conditions with cellulose as carbon source and we tested for a potential relevance of MAPkinases in the strong down-regulation of cellulases in light.

We observed that lack of *tmk3* in the genome virtually abolished specific cellulase activity in darkness (Fig. 2B), which is in agreement with the strongly decreased biomass formation of Δ*tmk3* under these conditions (Fig. 1B). Due to the strong effect of TMK3 on cellulase regulation, chemotropic response to glucose and biomass formation upon growth on cellulose, we were interested whether the growth defect of Δ*tmk3* is a general phenomenon or conditions specific i.e. carbon source specific. Analysis of hyphal extension of Δ*tmk3* on malt extract medium (3% w/v) showed a colony size decreased by 48 + /− 1% (standard deviation of 3 biological replicates), on carboxymethylcellulose the decrease was considerably stronger with 86 + /− 1% and on glucose Δ*tmk3* showed no growth after the 48 h in darkness of the experiment used in parallel for the other measurements. Consequently, the growth defect caused by the lack of TMK3 is obvious on all media used, albeit the extent of the retardation is dependent on the carbon source. The more severe growth defect on carboxymethylcellulose compared to the full medium (malt extract) is in agreement with the strong decrease of cellulase expression in Δ*tmk3*. The fact that Δ*tmk3* does not chemotropically react to glucose anymore, a degradation product of cellulose is in agreement with its growth defect on glucose, as it obviously as problems to sense it, which may well be connected to perturbed cellulase regulation and the subsequent glucose liberation intra- and/or extracellularly.

Deletion of *tmk1* caused increased cellulase activity and for Δ*tmk*2 we found a positive trend (Fig. 2B). In the wild-type QM6a, cellulase activity in light decreases to levels around or below the detection limit^79^, which did not change in deletion strains of *tmk1*, *tmk2* or *tmk3* (data not shown). Consequently, MAPkinases are not involved in the (posttranscriptional) mechanism responsible for the block of cellulase formation in light, although they do influence *cbh1* transcript abundance.

Transcript abundance of *cbh1*, the major cellobiohydrolase gene of *T. reesei*, correlated with the results for specific cellulase activity in darkness, with significantly increased *cbh1* levels in Δ*tmk2*, hence supporting the positive trend of cellulase activity in Δ*tmk2* (Fig. 2C). In light, *cbh1* transcript levels are decreased in all three MAPkinase mutants (Fig. 2D), reflecting a clear difference to the situation in darkness.

In darkness, transcript levels of the major cellulase transcription factor gene *xyr1* correlates with those of *cbh1* (Fig. 2E), which was shown for other conditions previously^86^. Also for *xyr1*, the situation is different in light (Fig. 2F), in that the correlation with *cbh1* was not observed and in contrast to the down-regulation of transcript levels of *cbh1* in Δ*tmk2*, *xyr1* transcript levels follow the up-regulation as seen in *cbh1* and *xyr1* in this strain in darkness. Therefore, it is tempting to speculate that TMK1 and TMK3, but not TMK2 are relevant for the function of XYR1 in cellulase regulation in light. Since XYR1 comprises MAPK phosphorylation sites^76^, this would not be without precedent.

In case of the carbon catabolite repressor gene *cre1*, we also found clear differences in gene regulation by TMK1, TMK2 and TMK3 in light and darkness (Fig. 2G,H). The lack of significant regulation of *cre1* in darkness does not indicate a relevance of MAPkinases for carbon catabolite repression at the level of modulation of transcript abundance of *cre1* (Fig. 2G). In light, *cre1* transcript abundance decreases in all three deletion strains (Fig. 2H), the relevance of which is difficult to interpret, due to the very low levels of expressed cellulases in light on cellulose.

### MAPkinases are involved in sorbicillin production

**Figure 3:**
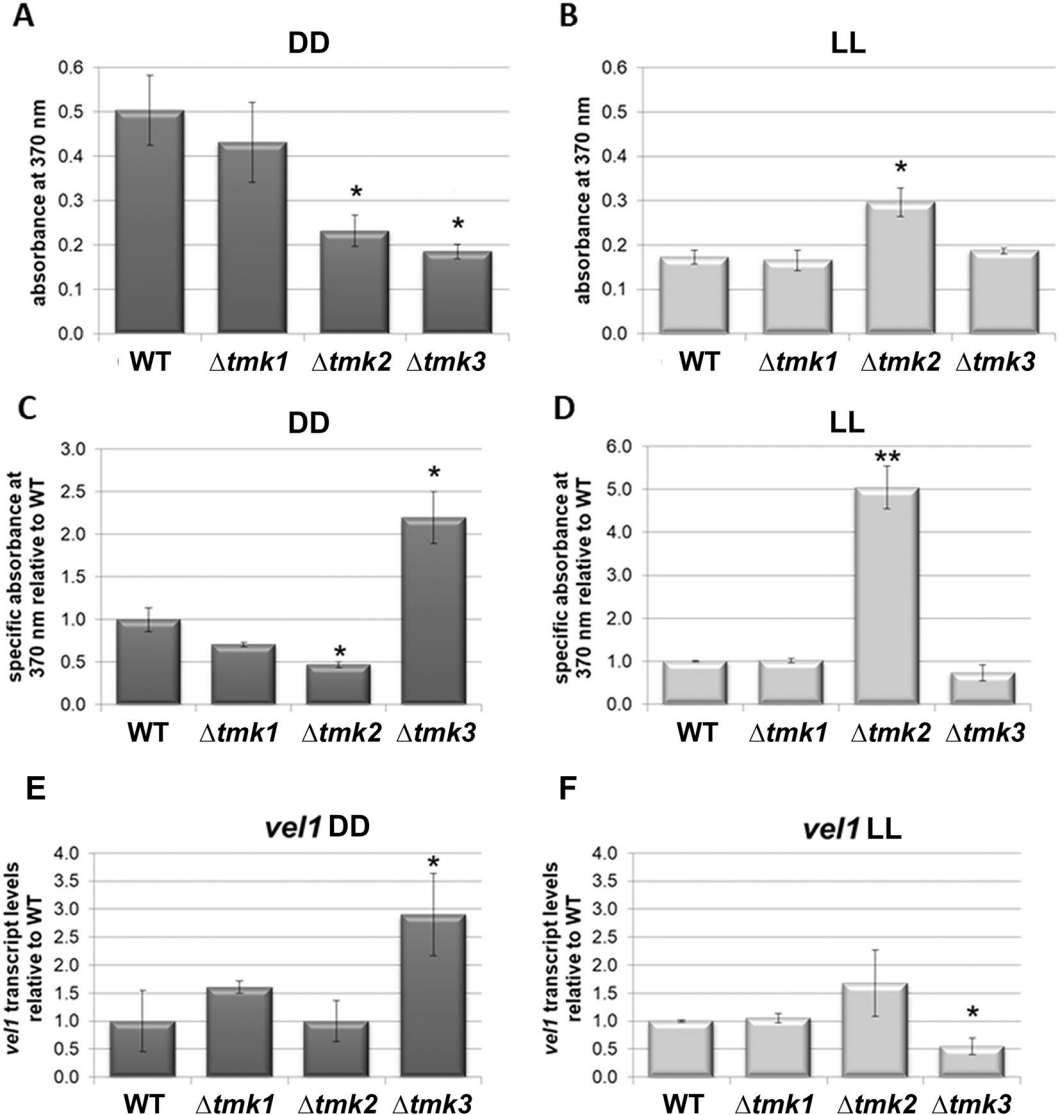
Relevance of MAPkinases for sorbicillin production and genes involved in secondary metabolism. (**A**,**B**) Evaluation of sorbicillin production as influenced by MAPkinases. Absorbances are shown for 370 nm, which is representative for sorbicillins^90^. (**C**,**D**) Specific sorbicillin abundance in supernatant as related to biomass formation upon growth on 1% cellulose in (**C**) constant darkness and (**D**) constant light. (**E**,**F**) Regulation of transcript abundance of *vel1* in MAPkinase mutant strains in (**E**) constant darkness and (**F**) constant light.

An involvement of MAPkinases of *T. reesei* in regulation of secondary metabolism has not been tested previously. Sorbicillin production is connected to the regulation of cellulase gene expression and carbon catabolite repression in *T. reesei*^50,87,88^. Therefore, we assessed this function with a photometric screening for yellow pigments representing mainly sorbicillin derivatives, which show a typical light absorbance maximum close to 370 nm. These compounds are biosynthetized by the products of the SOR secondary metabolite cluster^50,89,90^ upon growth on liquid media with cellulose as carbon source (Fig. 3A,B).

We found that both TMK2 and TMK3 positively influence sorbicillinoid production in darkness upon growth on cellulose (Fig. 3C), which correlates with the difference in biomass production in case of Δ*tmk3*. In light, the situation is reversed for TMK2 (Fig. 3D), which has a considerably negative effect on the production of sorbicillin derivatives. This prompted us to investigate a possible influence of MAPkinases on secondary metabolism in more detail.

### MAPkinases impact regulation of secondary metabolism

Among the most crucial regulators of secondary metabolism is VEL1, which regulates sexual development and secondary metabolism in *T. reesei*^91^, shows a regulatory interaction with the photoreceptor ENV1^92^ and is essential for cellulase gene expression^93^. Therefore, we asked whether the regulatory function of the MAPkinases might be connected to the role of VEL1 by testing transcript abundance of *vel1* in deletion strains of *tmk1*, *tmk2* and *tmk3*.

Indeed, we found a light dependent regulation of *vel1* in all MAPkinase mutants, with differential impacts either in constant light or in constant darkness (Fig. 3E,F). The regulation pattern of *vel1* did not correlate with production of sorbicillin derivatives (Fig. 3A,E) as the clear increase of *vel1* transcript abundance in Δ*tmk3* should rather result in an increased level of sorbicillinoid production in case of a direct correlation, which is not the case. Consequently, the regulatory impact of the MAPkinases on sorbicillin production is unlikely to be mediated by VEL1.

### MAPkinases are required for normal sexual development

**Figure 4:**
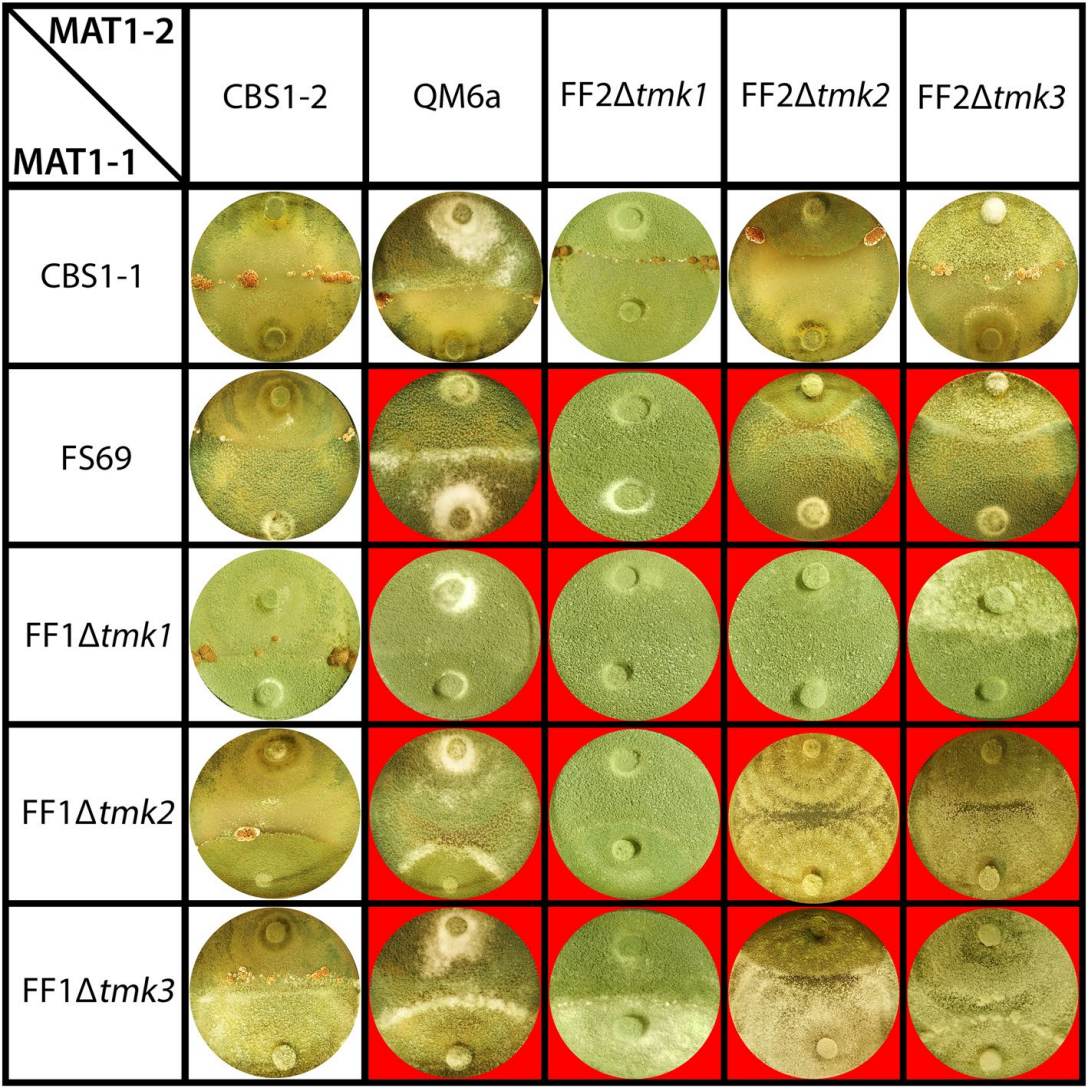
Involvement of MAPkinases in mating abilities. Sexual development of backcrossed MAPkinase mutant strains after 14 days grown in light cycles (12 h light, 12 h darkness) at 22 °C.

An involvement of MAPkinases in regulation of sexual development was shown previously in fungi. Since the parental strain QM6a is female sterile due to a defect in the MAPkinase scaffolding protein HAM5^64,65^, we outcrossed this defect by mating with the fully fertile QM6a derivative FF1. The resulting strains with fully fertile strain background were confronted under conditions favouring sexual development. All strains were able to form fruiting bodies with the fully fertile wild-type strains CBS999.97 MAT1-1 and CBS999.97 MAT1-2 (Fig. 4). However, none of the strains lacking a MAPkinase gene could mate with a female sterile strain of the respective compatible mating type (FS69 or QM6a) or with another strain lacking a MAPkinase. Therefore, we conclude that deletion of *tmk1*, *tmk2* or *tmk3* causes female sterility.

In homozygous crosses of strains lacking TMK2 or crosses between Δ*tmk2* and Δ*tmk3* of either mating type we observed a small but visible clearing zone. This finding suggests that the clear effects in regulation of secondary metabolism under different conditions by TMK2 and TMK3 also affect chemical communication and potentially cause a retardation of growth or decrease in aerial hyphae formation prior to contact. The minor effects of TMK1 on secondary metabolism are unlikely to be relevant for chemical communication. However, it has to be noted that for example fatty acid derived secondary metabolites would not be detected in our assay and hence we cannot fully exclude an influence of TMK1 on certain compounds not observed here.

### MAPkinases contribute to regulation of chemical communication

Secondary metabolite production changes under fermentative conditions in *T. reesei*, which was also shown for sorbicillinoids^94,95^, which are responsible for the yellow coloration of liquid and solid media inoculated with *T. reesei* wild-types^89,90^. The involvement of TMK2 and TMK3 in regulation of secondary metabolism and the relevance of all three MAPkinases for sexual development prompted us to assess their role in chemical communication under conditions facilitating mating.

**Figure 5:**
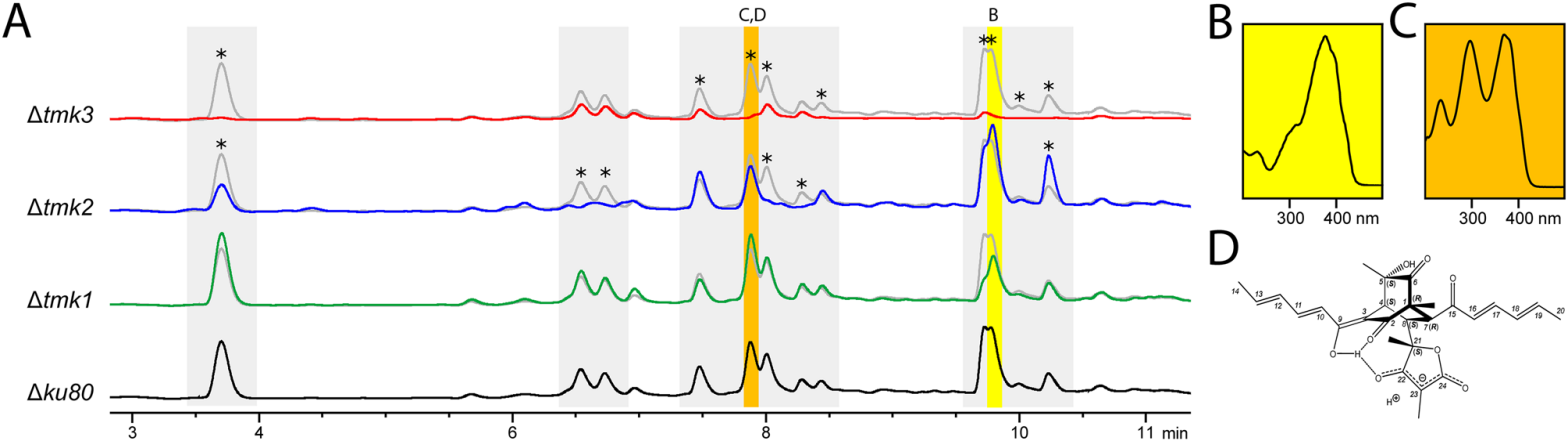
HPLC analysis of MAPK deletion mutants and identification of sorbicillin derivatives. (**A**) Chromatograms of wild-type (*Δku80*) and MAPkinase deletion mutants (Δ*tmk1-3*) at 230 nm. Wild type profile is shown in grey for better comparission. Asterisks indicate strongly regulated peaks. Trichodimerol is highlighted in yellow and (21*S*)-bisorbibutenolide in orange. (**B**) UV-spectrum of trichodimerol. (**C**) UV-spectrum of (21*S*)-bisorbibutenolide. (**D**) *(21S)-bisorbibutenolide*^98^. Numbering of protons and carbons is shown in Fig. 5D and in agreement with those used previously^98^.

Our analyses showed that TMK1 is required for production of at least one metabolite, which is also decreased upon lack of TMK3. Deletion of *tmk2* further resulted in a shift of abundance of certain secondary metabolites (Fig. 5). The most striking effect was found for Δ*tmk3* (Fig. 5A) revealing that in this strain the production of all compounds detected in the wild-type was downregulated or abolished. Using a reference compound^95^, we could identify the sorbicillin derivative trichodimerol that is strongly regulated by TMK3 (Fig. 5A and Figure S1). Hence, the hypothesis that MAPkinases contribute to regulation of chemical communication of *T. reesei* by secreting (secondary) metabolites to the environment is well supported. However, although a correlation of defects in secondary metabolite secretion with perturbed mating behavior was reported previously^91,94^, the precise role of these secondary metabolites in initiation of sexual development still remains to be clarified.

Considering the results for growth in liquid media with cellulose as carbon source, we conclude that MAPkinases represent important signaling cascades, differentially integrating signals with varying relevance upon growth on different carbon sources, on surfaces or submerged and in dependence of light.

### MAPkinases regulate production of trichodimerol (21S)-bisorbibutenolid

Besides trichodimerol as product of the SOR cluster, also several other compounds showed alterations in one or more MAPkinase deletion strains. Hence, we were interested in the nature of these compounds and aimed at isolation and structural elucidation of one strongly regulated and hence the most interesting changing peak. Due to the complexity of different structures of sorbicillinoids, which nevertheless show similar UV spectra, we aimed to purify a compound of interest to enable unequivocal assignment of the structure.

2.8 The yellow color of the compound selected for detailed anaysis revealed that it is likely to be a sorbicilliniod and mass spectrometry indicated a similarity with bisorbibutenolide, which required more indepth investigation for confirmation. (21*S*)-bisorbibutenolide (Fig. 5B–D), isolated from extract of *T. reesei*, shows in HR-ESI-TOF-MS in negative ionization a deprotonated molecular ion [M–H]^−^ of *m/z* 495.2033, and a [M + Na]^+^ of *m/z* 519.1980 in positive ionization mode. This correlates quite well with the calculated [M–H]^−^ of *m/z* 495.2024 and [M + Na]^+^ of *m/z* 519.1989 of the molecular formula C_28_H_32_O_8_. 1D and 2D NMR measurements led to a total number of six methyl-, zero methylen-, eleven methine groups and eleven quaternary carbon atoms resulting in three additional non carbon bound protons. Further investigations of the UV and NMR spectroscopic as well as MS spectrometric data imply a molcular structure of an unsymetric dimer of sorbicillinol.

The central moiety of this dimer is identified as a bicyclo[2.2.2]octane sleketon. This structure can be determined in HMBC by the ^2^*J*_C–H_ and ^3^*J*_C–H_ couplings of protons in its positions 4, 7 and 8 as well as of the protons in two methyl substituents in positions 1 and 5 (Fig. 5B). Namely, the methyl group at position 1 shows couplings to the carbons C-1, C-2, C-6 and C-7 while the methyl group at position 5 shows couplings to C-4, C-5 and C-6. Protons H-4, H-7 and H-8 each show eight or nine C-H long range couplings to the corresponding carbons via two or three covalent bonds, respectively (Figure S2). Some of these couplings even reach to carbon atoms in substituents which are bound to the bicyclo[2.2.2]octane sleketon. Additionally, chemical shifts of δC 210.7 and 197.4 as well as the multiplicities of carbons C-2 and C-6 indicate the presence of ketone functionalities in these positions. Furthermore, the chemical shift and the multiplicity of C-5 indicate that attached apart from the methyl group there is a hydroxy group bound in this position.

A (*E*,*E*)-hexa-2,4-dienoyl (sorbyl) substituent is attached in position 7 to the bicyclo[2.2.2]octane. This substituent can be identified by ^3^*J*_H–H_ couplings in COSY (Figure S3) as well as in HSQC by the ^2,3^*J*_C–H_ couplings within this moity and to the methine group in position 7 (Figure S2). The *E* configurations of both double bonds result in particular from the quite large ^3^*J*_H–H_ coupling constants between the sp^2^ hybridised methin groups. An second (*E*,*E*)-hexa-2,4-dienoyl substituent can be identified to be bound in position 3. However, this moiety is predominately present as enol tautomer between C-9 and C-3, which emerges of the chemical shifts and multiplicities of these two carbon atoms. The presence of these two diene conjugated carbonyl chromophores can be confirmed by UV absorption at 372 nm (Figure S4). Furthermore, an enolized 3-oxo-2,4-dimethylbutanolide ring is bound to C-8. The carbon skeleton of this moiety can be identified by the ^2^*J*_C–H_ and ^3^*J*_C–H_ couplings of the protons in methyl groups bound to C-21 and C-23. The chemical shifts of C-22, C-23 and C-24 (δC 188.8, 92.3 and 180.2, respectively) further clearly indicate the enolization in this structural moiety.

The relative stereochemistry of (21*S*)-bisorbibutenolide was determined using NOEs recorded in the NOESY spectum (Figure S5). The stereochemistry at positions 4, 5, 7 and 8 in the bicyclo[2.2.2]octane sleketon can especially be explained by NOEs between the CH_3_ group at C-5 and the protons H-10 and H-11 as well as by the missing NOEs from this methyl group to H-7 and H-8. Furthermore, H-8 shows an NOE to H-16 as well as H-7 has an NOE to the methyl group at position 21. The absolute stereochemistry was deduced on the stereochemistry of *S*-sorbicillinol, which is yet only repored enantiomer of this natural product^96^ (Scifinder, 2022). It results in the (1*R*,4*S*,5*S*,7*R*,8*S*)-bisorbibutenolide for the stereocenters in the central moiety (Fig. 5C), which are in agreement with those reported earlier^97,98^ for the same molecular structure. Furthermore, the stereochemistry at position 21 in the butanolide moiety was determined with regard to Maskey et al.^98^. They have shown that an 21*S* configuration causes the deprotonation of the OH group in position 22 with a concomitant enolisation of C-22, C-23 and C-24. This is caused by a spatial proximity of the deprotonated hydroxy group at C-22 to the hydroxy group at C-9 as well as to the ketone at C-3. In case of a 21*R* configuration, such deprotonation occures to a significantly lesser extent, since the described spatial proximity between C-3, C-9 and C-22 is not possible.

Overall, the structure is those of (21*S*)-bisorbutenolide, which is shown in Fig. 5D. All recorded spectroscopic data are summarized in section “Materials and Methods” and the spectra are shown in the Supplementary Material (Figures S2–S11). These data are consistent with those reported by Maskey et al.^98^ for (21*S*)-bisorbutenolide as well as well as with those reported by^97^ for the structurally identical “trichotetronine”. Thus, we assume that all three independently determined structures are identical.

## Discussion

**Figure 6:**
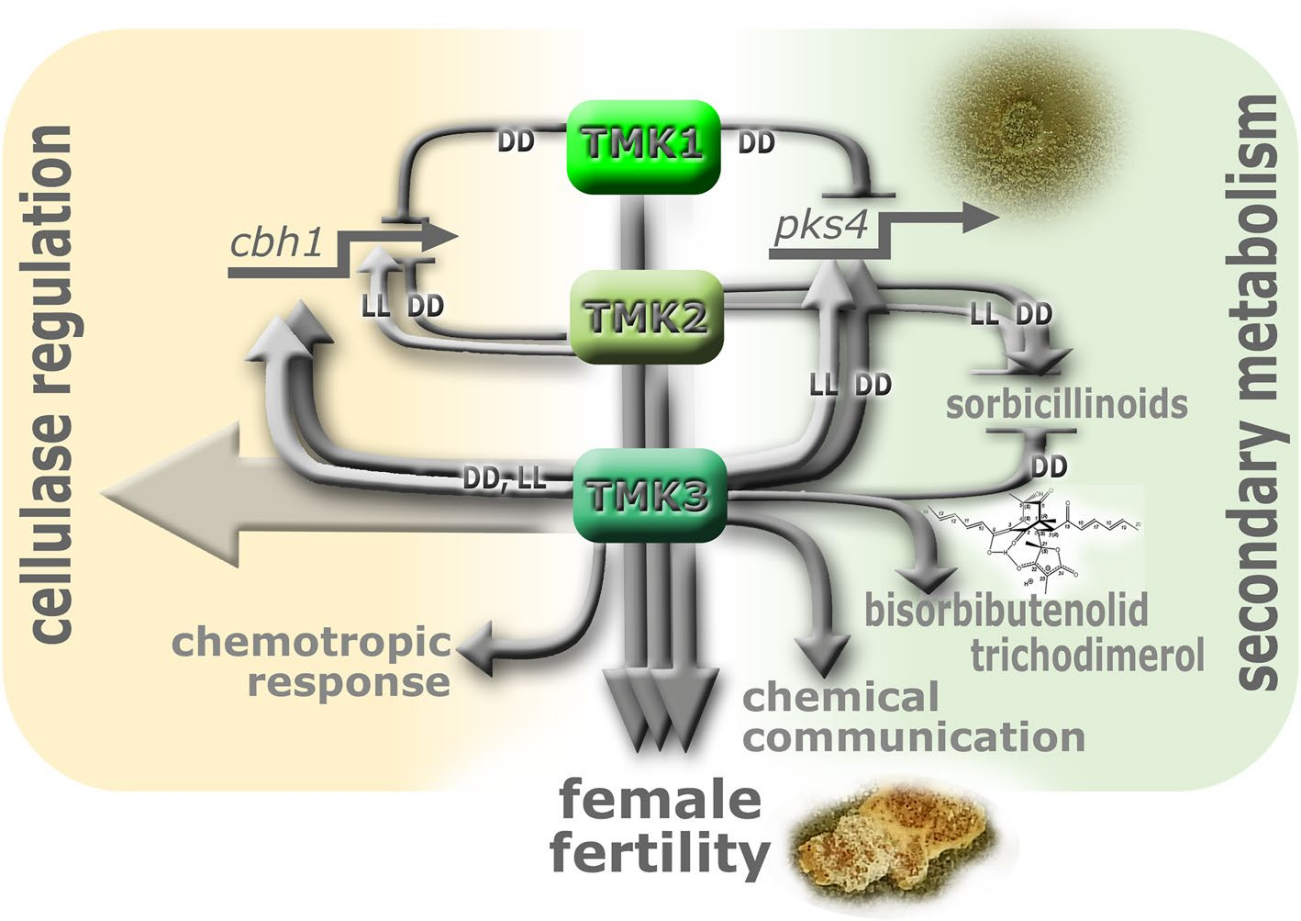
Schematic representation of the involvement of the MAPKinases, TMK1, TMK2 and TMK3 in sexual development (female fertility), cellulase regulation and secondary metabolism in constant light (LL) and constant darkness (DD). The figure was designed in Adobe Photoshop CS6 by MoS.

Fungi have to react to multiple environmental cues to succeed in competition in order to balance resources between investment in biomass formation and colonization, reproduction and warfare—production of secondary metabolites to defend nutrients, mating partners and reproductive structures. Our study revealed that the MAPkinase pathways of *T. reesei* are central to regulation of these tasks, as they differentially integrate signals and coordinately rather than separately modulate their output pathways (Fig. 6). The different functions, which TMK1, TMK2 and TMK3 assume are all influenced by light. This is in perfect agreement with the crucial functions of their homologues in light response and circadian clocks in other fungi. Importantly, the MAPkinase pathway acts downstream of the circadian clock and hence also of the photoreceptor complex members as its core components^28,99^. Thereby, the MAPkinases obviously provide important information on the environment which are integrated with the light signals perceived by photoreceptors to achieve an appropriate response in light or darkness.

For TMK1 we see a small, but significant increase in specific cellulase activity in darkness and a corresponding trend in slightly elevated *cbh1* and *xyr1* transcript levels, while in light *cbh1* transcript levels decrease, which may have contributed to the lack of detection of an effect of TMK1 in previous work^75^.

TMK2 negatively influences cellulase expression upon growth on wheat bran combined with Avicel. However, biomass formation of this strain is unclear and data on specific activity are not available in this study^74^. Deletion of *tmk2* caused decreased growth in the presence of lactose and glucose, but not glycerol in *T. reesei*^77^. We could now confirm the negative impact of TMK2 on cellulase regulation in *T. reesei* upon growth on cellulose. This regulatory effect is reflected in an increase of transcript abundance of *cbh1* and *xyr1* as well as a positive trend in specific cellulase activity in Δ*tmk2*. The previously detected only minor effect of TMK2 on *cbh1* transcript abundance may be due to the uncontrolled light conditions during cultivation: Since we observed a clear increase of *cbh1* in Δ*tmk2* in darkness and a decrease in light, what previously was found, may well be a mixture of these effects.

In case of TMK3 our results for cellulase regulation are in agreement with previous data^76^, although also here the regulation pattern we observed is more severe, with activity and transcript levels barely detectable anymore. Again, random light pulses during cultivation and harvesting may have alleviated the strongly decreased values we found.

MAPkinases are well known to act at higher levels of the signaling cascade, above the transcription factors of the downstream pathways, which may be impacted directly by phosphorylation or indirectly be regulation of positive or negative factors influencing them. However, a potential feedback regulation acting via a nutrient sensing pathway might still influence regulation of MAPkinase genes at the transcriptional level. We therefore checked available transcriptome data from comparable conditions for indications if such a feedback might exist^50,100–102^, but since we did not find significant regulation of *tmk1, tmk2* or *tmk3* in these data, we conclude that this is not the case.

Interestingly, in *N. crassa* the OS pathway, corresponding to the Hog1-pathway in yeast and comprising a homologue of TMK3 has no significant influence on cellulase production^103^, which is in contrast to our results.

In summary, our data obtained with experiments under controlled light conditions clearly show a light dependent regulatory function of all three MAPkinases on cellulase gene regulation and secreted cellulase activity, which is jeopardized by random light pulses.

The GPCR CSG1, which is essential for the chemotropic response of *T. reesei* to glucose^84^, was shown to be required for posttranscriptional regulation of cellulase gene expression^78^. Importantly, this GPCR is not related to other known glucose sensing GPCRs like GPR-1 in *N. crassa* or Gpa2 of *S. cerevisiae*^78^. In contrast, the function of CSG1 as a member of class XIII of GPCRs was for the first time characterized as posttranscriptional regulation of cellulases^78^. Here we found that also TMK3 is needed for the chemotropic response to glucose, although here, in contrast to the situation with CSG1^78^, not only cellulase activity, but also transcript abundance decrease strongly (Fig. 2B,C). Hence, we assume that perturbed chemotropic reaction to glucose does not necessarily correlate with diminished cellulase transcript abundance, but is likely to be important for regulating the amount of produced cellulases at different levels.

Interestingly, research with *F. oxysporum* showed a dependence of the chemotropic response to glucose on TMK1^22^, which we did not observe and the relevance of TMK3 on this process was not studied yet. Due to the different habitats and ecological functions of these two fungi—*F. oxysporum* being a plant pathogen and *T. reesei* mainly a saprotroph—glucose sensing may have a different relevance in these fungi. However, the widespread presence and conservation of MAPkinase pathways from yeast to man rather speaks against such a hypothesis and the reason for this discrepancy remains to be investigated.

We found that the glucose signal is transmitted via the class XIII GPCR CSG1, which is also essential for the chemotropic response to glucose^84^. Our results for TMK3 reveal, that this chemotropic response is not exclusively channeled throught the heterotrimeric G-protein pathway, but also through the MAPkinase pathway. Hence, a potential role of biased GPCR signaling^85^ in the chemotropic response to glucose is worth exploring in *T. reesei*.

Female sterility is defined as the inability to assume the female role during sexual development and can have diverse physiological reasons^104^ including a defect in hyphal fusion, for example due to mutations in the *ham5* gene^105,106^. In fungi like *N. crassa*, formation of protoperithecia is induced in the female strain prior to fertilization with conidia of the male strain to assess male and female fertility. In *T. reesei* this method is not applicable, because no growth condition is known under which such structures are formed. Consequently, tests for male or female fertility are performed by assessment of mating and fruiting body formation with strains comprising a female sterile strain background in addition to the deletion of the gene of interest or as mating partners^63^. Defects in sexual development due to lack of MAPkinases were shown for all three pathways in *N. crassa*^107^ as well as in other fungi. Sexual development is consistently impacted by all three MAPkinases in *T. reesei*, which are obviously responsible for the ability to mate with a partner having a defect in female fertility such as mutations in HAM5. HAM5 acts as a scaffolding protein for MAPkinase pathways and is crucial for their function^106^. Consequently, the phenotype we see upon deletion of *tmk1*, *tmk2* and *tmk3* is in agreement with the female fertility caused by the pathway involving HAM5, which is also responsible for the sexual defect of *T. reesei* QM6a^64,65,67^.

Since at least the TMK1 and TMK2 mutant strains in *S. macrospora* and *N. crassa* are fusion mutants as are those lacking HAM5^105,108^, it would not be without precedent if the sexual defect of the *T. reesei* MAPkinases were due to abolished ability of hyphal fusion in these strains as well.

Carbon catabolite repression was recently reported to be impacted by the high osmolarity MAPK pathway, which contributes to a protein complex regulating CreA cellular localization and dissociates upon addition of glucose^109^. In *N. crassa*, genetic and omics analyses showed that the MAPkinase pathway is not acting through the canonical carbon catabolite repressor CRE-1^103^. Hence, the minor changes in transcript abundance we found for regulation of *T. reesei cre1* by MAPkinases in light gives a hint to their relevance, but does not reflect the full mechanism of regulation, which may be considerably more significant at the protein- and interaction level also in *T. reesei*. However, the abolished chemotropic response to glucose in a strain lacking TMK3 suggests that the Hog pathway may be connected to glucose signal transmission also in *T. reesei*. Additionally, the differences between light and darkness we see in our experiments indicate that both conditions should be investigated in fungi to obtain a comprehensive picture.

As previously shown in *T. reesei*, interaction with potential mating partners of opposite mating types involves specifically changing secondary metabolite patterns^91,94^. We chose conditions enabling sexual development for our assay to enable conclusions as to altered chemical communication by strains lacking one of the MAPkinases. Among the compounds regulated via TMK3 is the sorbicillinoid bisorbibutenolide^110^. Bisorbibutenolid (or bislongiquinolide) deters the aphid *Schizaphis graminum* from feeding^111^ and showed significant growth inhibitory activity against cancer cell lines through cytostatic and not cytotoxic effect^112^. The production of bisorbibutenolide is hence likely to be aimed at fending off competitors, which is in agreement with findings in *T. atroviride* on larvae preferentially feeding on *tmk3* mutants^73^. However, the SOR cluster , which is mainly responsible for sorbicillinoid production in *T. reesei*, was acquired through lateral gene transfer and is subject to strong evolutionary selection^113^. This cluster is not present in *T. atroviride* and consequently, a conservation of this phenomenon between *T. reesei* and *T. atroviride* remains to be shown.

## Materials and methods

### Strains and cultivation conditions

The wild-type strain used in this study is QM6aΔ*ku80*^50^ (deficient in non-homologous end joining). For analysis of gene regulation, enzymatic activity and biomass formation by TMK1, TMK2 and TMK3 strains were grown in liquid cultivation in constant light (white light; 1700 lx) or constant darkness, 200 rpm and 28 °C for 96 h. Before inoculation, strains were grown on 3% (w/v) malt extract (MEX) agar plates in constant darkness for 14 days (to exclude influences by the circadian rhythm). For liquid culture 10^9^ conidia/L were inoculated in Mandels Andreotti minimal medium^114^ with 1% (w/v) microcrystalline cellulose (Alfa Aesar, Karlsruhe, Germany) as carbon source, 5 mM urea and 0.1% peptone to induce germination. After 96 h, mycelia and supernatants were harvested, for the constant darkness cultures only a very low red safety light (darkroom lamp, Philips PF712E, red, 15W) was used as single light source.

### Construction of recombinant strains

Deletion of *tmk1*, *tmk2* and *tmk3* was done in QM6aΔ*ku80* following the procedure as described previously^80^ with the hygromycin (*hph*) marker cassette constructed by yeast recombination of the 1 kb flanking regions up- and downstream of the gene of interest and the *hph* marker. Transformation was done by protoplasting and 50 µg/mL hygromycin B as selection reagent (Roth, Karlsruhe, Germany)^115^. Protoplasts were isolated three to six days after transformation and subjected to a minimum of two rounds of single spore isolation. Successful deletion was confirmed by the absence of the gene by PCR (Table S1). All three mutants were confirmed to only have a single integration of the deletion cassette by copy number determination^102^.

### Crossing and selection for fully fertile progeny for assessment of sexual development

All crosses for the analysis of sexual development were performed on 60 mm 2% MEX agar plates at 22 °C and 12 h light–dark cycles as previously described^116^. To obtain progeny carrying the deletion in both mating types with a functional *ham5* gene, the mutant strains in the QM6a (MAT1-2, defective *ham5* copy) background were crossed with the female fertile strain FF1 (MAT1-1, functional *ham5* copy). The FF1 strain was obtained from backcrossing the female fertile strain CBS999.97 (described in detail previously^65^) 10 times with QM6a to acquire sexual fertility while retaining the QM6a phenotype^91^. Ascospore derived progeny were analyzed for the presence of gene deletion and mating type by PCR (Table S1). The functionality of the *ham5* gene was confirmed by high resolution melt curve (HRM) analysis, performed as described previously^117^.

### Isolation of nucleic acids and RTqPCR

Isolation of RNA was done from mycelia from liquid culture using the Qiagen RNeasy Plant mini kit following the manufacture’s guidelines. After DNase digest (ThermoFisher) of 1 µg total RNA and cDNA synthesis (GoScript reverse transcriptase, Promega, Madison, WI, USA), RT-qPCR was performed using the GoTaq® qPCR Master Mix (Promega) as previously described with *sar1* as reference gene and other primers listed in Table S1^94,118^. For RT-qPCR three biological and three technical replicates were considered, for *cbh1*, twice three technical replicates were included and for the analysis CFX maestro analysis software was used. Isolation of DNA for mutant and progeny screening, was done following the rapid minipreparation protocol for fungal DNA as described previously^119^.

### Analysis of enzyme activity and biomass formation

Enzymatic activity was measured from supernatants of liquid cultures using the CMC-cellulose kit (S-ACMC-L Megazyme) measuring endo-1, 4-ß-D-glucanases. For specific cellulase activities, the activities were correlated with the biomass produced which was determined from frozen mycelia in the presence of insoluble cellulose^45^. Shortly, mycelia were frozen in liquid nitrogen and ground with pestle and mortar before sonification and incubation in 0.1 M NaOH to break up cells. The freed protein content was measured using the Bradford method.

### Chemotropic response assay

Analysis of chemotropism assay was done essentially as described previously^22^ except that the water agar was supplemented with 0.0025% peptone as optimized previously^84^. The chemoattractant (1% glucose) was applied onto the plates in comparison with water as a control on the opposite side. The orientation of germ tubes was determined under the microscope (VisiScope TL524P microscope; 200 × magnification) and chemotropic indices calculated from a minimum of 3 biological replicates, counting a minimum of 400 germ tubes per plate, as previously described^22^.

### Photometric analysis of sorbicillinoid production

Supernatants of liquid cultivation were centrifuged for 5 min at 10.000 g to remove residual cellulose and absorbance at 370 nm indicative yellow sorbicillinoids were measured from biological triplicates.

### Isolation of (21S)-bisorbibutenolide

The dry crude extract (350 mg) was dissolved in 2 mL pure methanol (MeOH) and the obtained suspension centrifuged ar 14,000 rpm for 3 min. The supernatant was subsequently subjected to column chromatography over Sephadex LH20 eluted isocratically with pure MeOH. A total of 30 fractions á 5 mL were collected. Fractions 17 to 21 were pooled (11.3 mg) and finally purified by preparative thin layer chromatography (precoated glass plates, silica gel 60, F_254_, 0.25 mm thickness) developed in CHCl_3_/MeOH (95:5). This step afforded 4.3 mg of (21*S*)-bisorbibutenolide. All separation steps were monitored by HPLC.

### Secondary metabolite analysis by HPLC

For the extraction of secondary metabolites, strains were grown on 3% malt extract medium in constant darkness for 14 days. For each strain three biological replicates were used. For each sample, two agar plugs of 1,8 cm^2^ were taken from 3 plates. Agar plugs were collected in 15 mL tubes and 3 mL of 50% acetone in water (v/v) was added and put into an ultrasonic bath for 15 min for better dilution. Subsequently 1 mL of chloroform was added. Tubes were then centrifuged at 4 °C at 1000 g for 1 min for phase separation. The organic phase was transferred to a glass vial and chloroform extraction was repeated twice before the vials were left for evaporation over night. The dry extracts were redissolved in 140 μL methanol and stored in glass vials at − 20 °C before analysis.

Analytical HPLC measurements were performed on Agilent 1100 series coupled with UV-diode array detection at 230 nm and a Hypersil BDS column (100 × 4 mm, 3 µm grain size). An aq. buffer (15 mM H3PO4 and 1.5 mM Bu_4_NOH) (A) and MeOH (B) was used as eluents. The following elution system was applied: From 55–95% B within 8 min, and 95% B was kept for 5.0 min, with a flow rate of 0.5 mL min^−1^. The injection volume was 5.0 µL.

### Statistics

Statistical significance was evaluated by the t-test in R-studio (compare means, ggpubr version 0.4.0) **p value < 0.01, *p value < 0.05. At least three biological replicates were considered in every assay.

### NMR spectroscopy

For NMR spectroscopic measurements (21*S*)-bisorbibutenolide was dissolved in CD_3_OD (~ 4.2 mg in 0.7 mL) and transferred into 5 mm high precision NMR sample tubes. All spectra were measured on a Bruker DRX-600 at 600.18 MHz (^1^H) or 150.91 MHz (^13^C) and performed using the Topspin 3.5 software. Measurement temperature was 298 K ± 0.05 K. 1D spectra were recorded by acquisition of 64 k data points and Fourier transformed spectra were performed with a range of 7200 Hz (^1^H) and 32,000 Hz (^13^C), respectively. To determine the 2D COSY, TOCSY, NOESY, HMQC, and HMBC spectra 128 experiments with 2048 data points each were recorded, zero filled and Fourier transformed to 2D spectra with a range of 6000 Hz (^1^H) and 24,000 Hz (HSQC) or 32,000 Hz (HMBC) (^13^C), respectively. Residual CD_2_HOD was used as internal standard for ^1^H NMR measurements (δH 3.34) and CD_3_OD for ^13^C NMR measurements (δC 49.0).

### Mass spectrometry

Mass spectra were measured on a high resolution time-of-flight (hr-TOF) mass spectrometer (maXis, Bruker Daltonics) by direct infusion electrospray ionization (ESI) in positive and negative ionization mode (mass accurancy +/− 5 ppm). TOF MS measurements have been performed within the selected mass range of *m/z* 100–2500. ESI was made by capillary voltage of 4 kV to maintain a (capillary) current between 30 and 50 nA. Nitrogen temperature was maintained at 180 °C using a flow rate of 4.0 L min^−1^ and the N_2_ nebulizer gas pressure at 0.3 bar.

### Spectroscopic data for (21S)-bisorbibutenolide^98^

UV_max, MeOH_, 234, 298, 372 nm; HR ESI-MS *m/z* 495.2033 [M–H]^−^ (calcd for C_28_H_31_O_8_^−^, 495.2024), *m/z* 519.1980 [M + Na]^+^ (calcd for C_28_H_32_O_8_Na^+^, 519.1989); ^1^H NMR (600 MHz, CD_3_OD): δ_H_ = 7.35 (1H, dd, *J* = 14.7, 11.7 Hz, H-11), 7.26 (1H, dd, *J* = 11.8, 11.0 Hz, H-17), 6.41 (1H, m, H-19), 6.40 (1H, m, H-13), 6.38 (1H, m, H-12), 6.37 (1H, m, H-18), 6.32 (1H, d, *J* = 14.7 Hz, H-10), 6.16 (1H, d, *J* = 11.8 Hz, H-16), 3.41 (1H, m, H-7), 3.35 (1H, m, H-4), 3.17 (1H, m, H-8), 1.89 (3H, d, *J* = 6.7 Hz, H-14), 1.88 (3H, d, *J* = 6.7 Hz, H-20), 1.42 (3H, s, CH_3_-23), 1.35 (3H, s, CH_3_-21), 1.18 (3H, s, CH_3_-5), 0.94 (3H, s, CH_3_-1); ^13^C NMR (150 MHz, CD_3_OD): δ_C_ = 210.7 (s, C-6), 203.9 (s, C-15), 197.4 (s, C-2), 188.8 (s, C-22)*, 180.2 (s, C-24)*, 169.4 (s, C-9), 148.0 (d, C-17), 144.8 (d, C-19), 143.1 (d, C-11), 140.3 (d, C-13), 132.4 (d, C-18), 131.7 (d, C-12), 128.9 (d, C-16), 119.2 (d, C-10), 110.4 (s, C-3), 92.3 (s, C-23), 85.0 (s, C-21), 75.9 (s, C-5), 63.8 (s, C-1), 51.8 (d, C-7), 43.9 (d, C-8), 43.7 (d, C-4), 24.0 (q, CH_3_-5), 23.2 (q, CH_3_-21), 19.0 (q, C-20), 18.9 (q, C-14), 11.4 (q, CH_3_-1), 6.4 (q, CH_3_-23); * determined via HMBC.

Numbering of protons and carbons is shown in Fig. 5D and in agreement with those used previously^98^. All data as well as the naming of the compound are in agreement with those reported earlier for this compound^97,98^, (there named as “trichotetronine”). It should be noted that the naming of this compound, particularly with regard to the stereochemistry at position 21, as well as of structurally and biosynthetically closely related compounds are not entirely consistent throughout the entire literature. 1D and 2D NMR spectra are shown in Figures S2, S3 and S5–S9, HR ESI MS spectra (pos. and neg. mode) are shown in Figure S10,S11, and chromatogram as well as UV spectrum are shown in Figure S4.

## Supplementary Information

Supplementary Information.

## Data Availability

The datasets generated and analysed during the current study are available from the corresponding author on reasonable request.
