# Supplementary material for: MAPkinases regulate secondary metabolism, sexual development and light dependent cellulase regulation in Trichoderma reesei
Source: Sci Rep. 2023 Feb 2;13:1912. doi: 10.1038/s41598-023-28938-w (PMC9894936; doi:10.1038/s41598-023-28938-w)
Supplement: Supplementary file 1 — Supplementary Information. [file 41598_2023_28938_MOESM1_ESM.docx]

**MAPkinases regulate secondary metabolism, sexual development and light dependent cellulase regulation in *Trichoderma reesei***

Miriam Schalamun^1^, Sabrina Beier^1^, Wolfgang Hinterdobler^1,2^, Nicole Wanko^1^, Johann Schinnerl^3^, Lothar Brecker^4^, Dorothea Elisa Engl^4^ and Schmoll Monika^1,5^*

^1^*AIT Austrian Institute of Technology GmbH, Center for Health and Bioresources, Bioresources Unit, Konrad Lorenz Strasse 24, 3430 Tulln*

*^2^ MyPilz GmbH, Wienerbergstrasse 55/13-15, 1120 Vienna, Austria*

*^3^Chemodiversity Research Group, Department of Botany and Biodiversity Research, University of Vienna, Rennweg 14, 1030 Vienna, Austria*

*^4^ Department of Organic Chemistry, University of Vienna, Währinger Strasse 38, A-1090 Vienna, Austria*

*^5^ University of Vienna, Department of Microbiology and Ecosystem Science, Division of Terrestrial Ecosystem Research, Djerassiplatz 1, 1030 Vienna, Austria*

**Supplementary material**


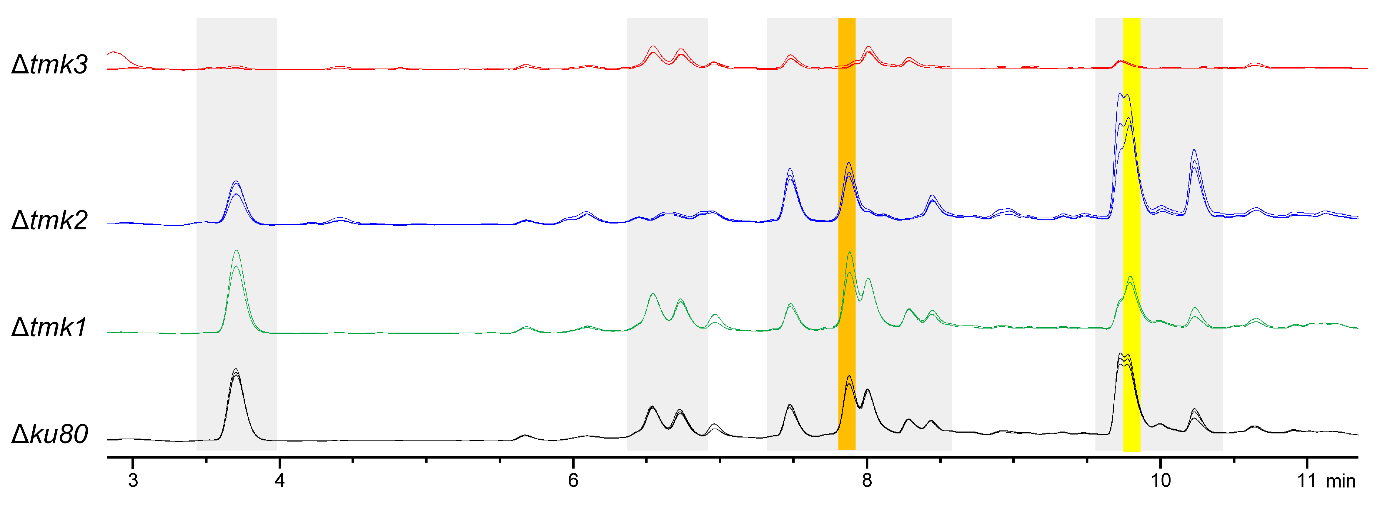


**Supplementary Figure S1.** HPLC analysis of MAPK deletion mutants and identification of sorbicillin derivatives. Chromatograms of biological replicates of wild type (Δ*ku80*) and MAPkinase deletion mutants (Δtmk1-3) at 230 nm. For Δ*tmk1* only two replicates were used for analysis.

**
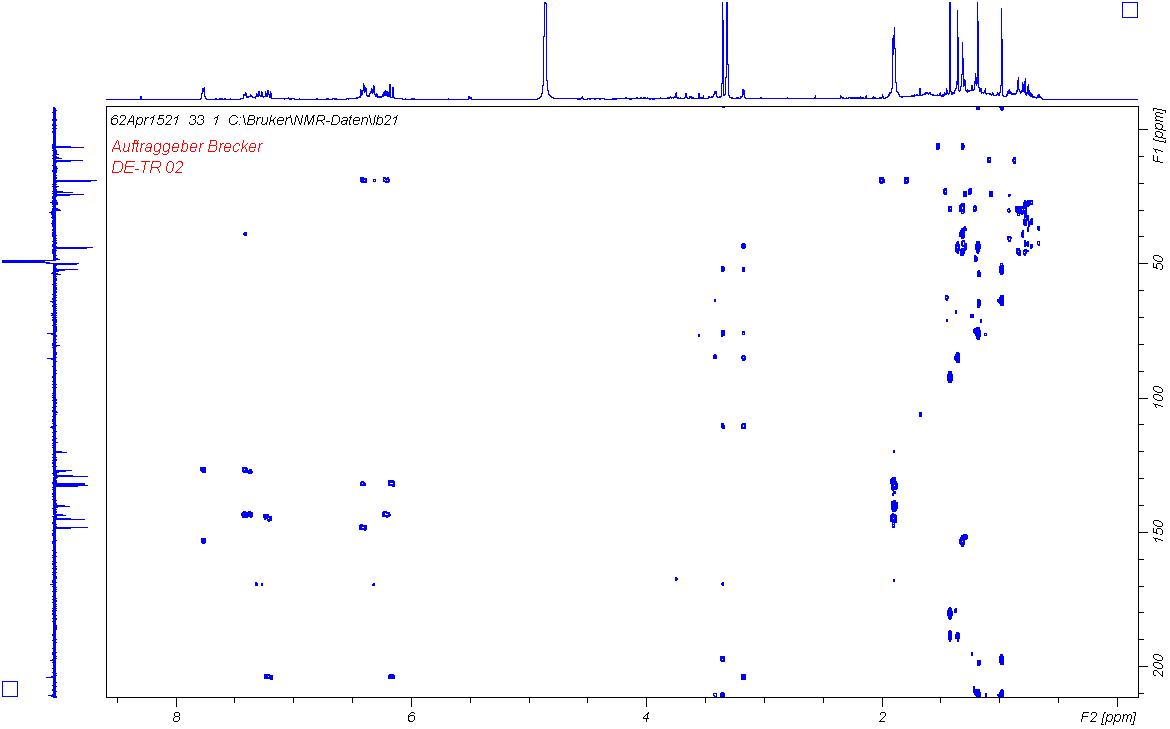
**

**Supplementary Figure S2.** HMBC of (21*S*)-bisorbibutenolide in CD_3_OD.

**
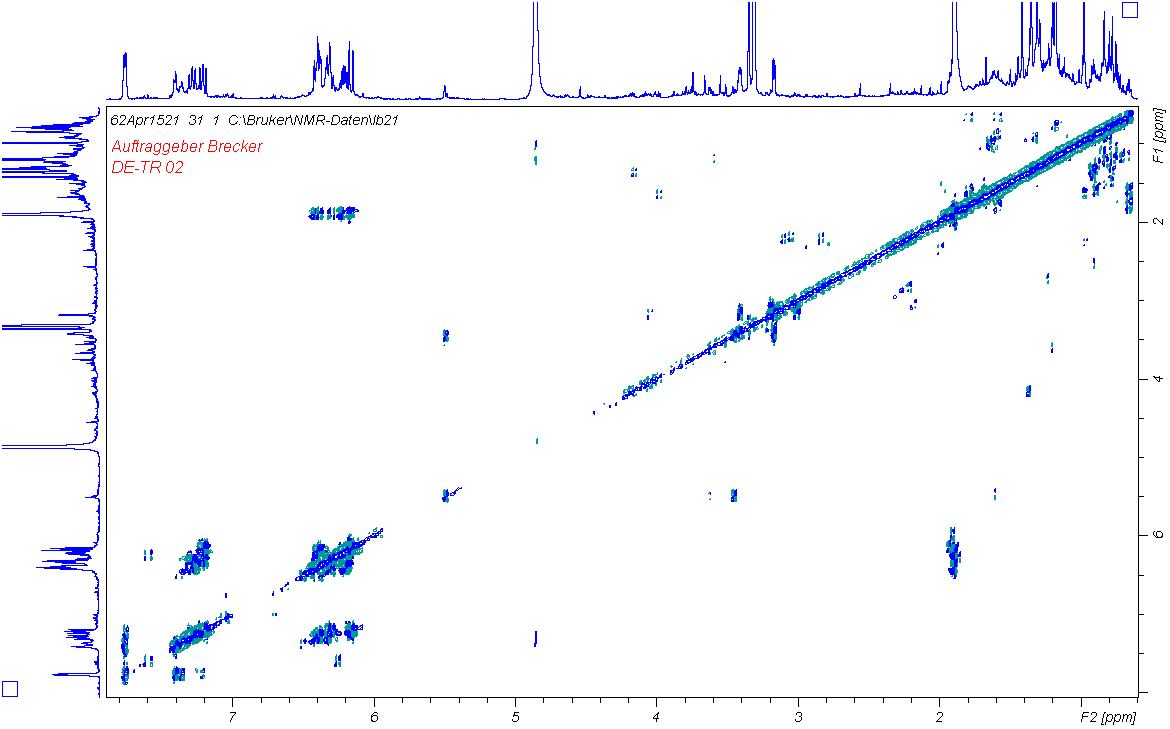
**

**Supplementary Figure S3.** COSY of (21*S*)-bisorbibutenolide in CD_3_OD.

**Supplementary Figure S4**. Chromatogram and UV/Vis spectrum of (21*S*)-bisorbibutenolide.

**
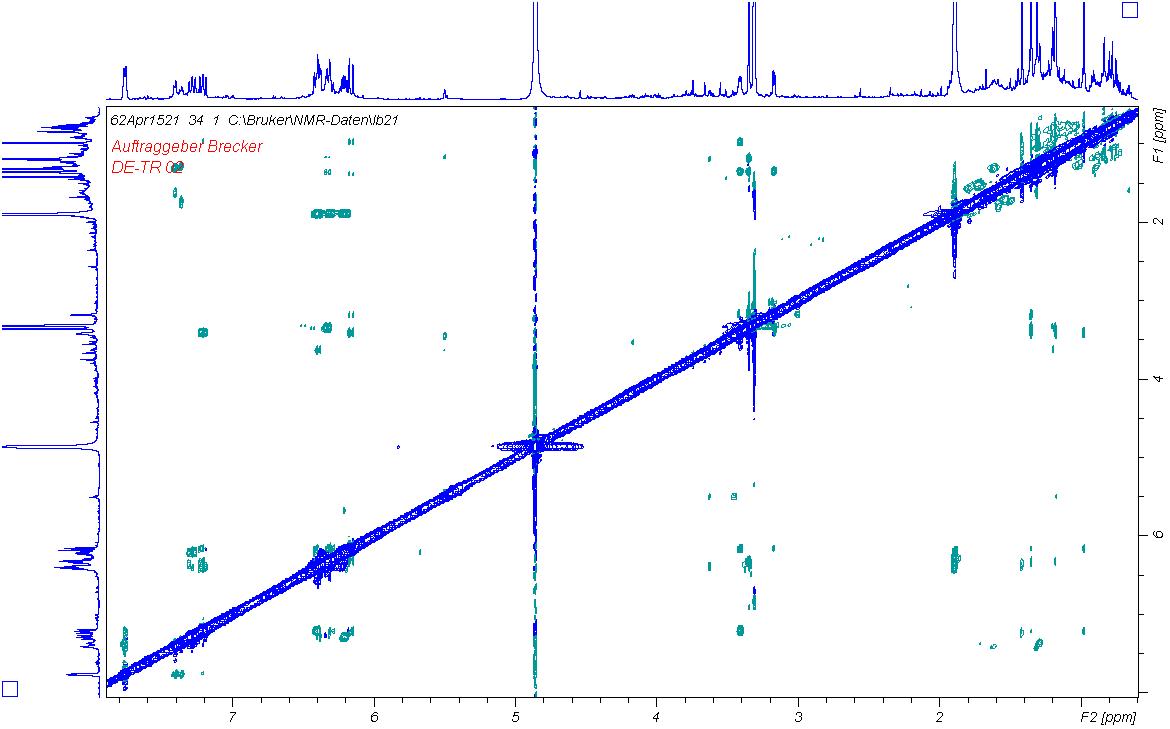
**

**Supplementary Figure S5**. NOESY of (21*S*)-bisorbibutenolide in CD_3_OD.


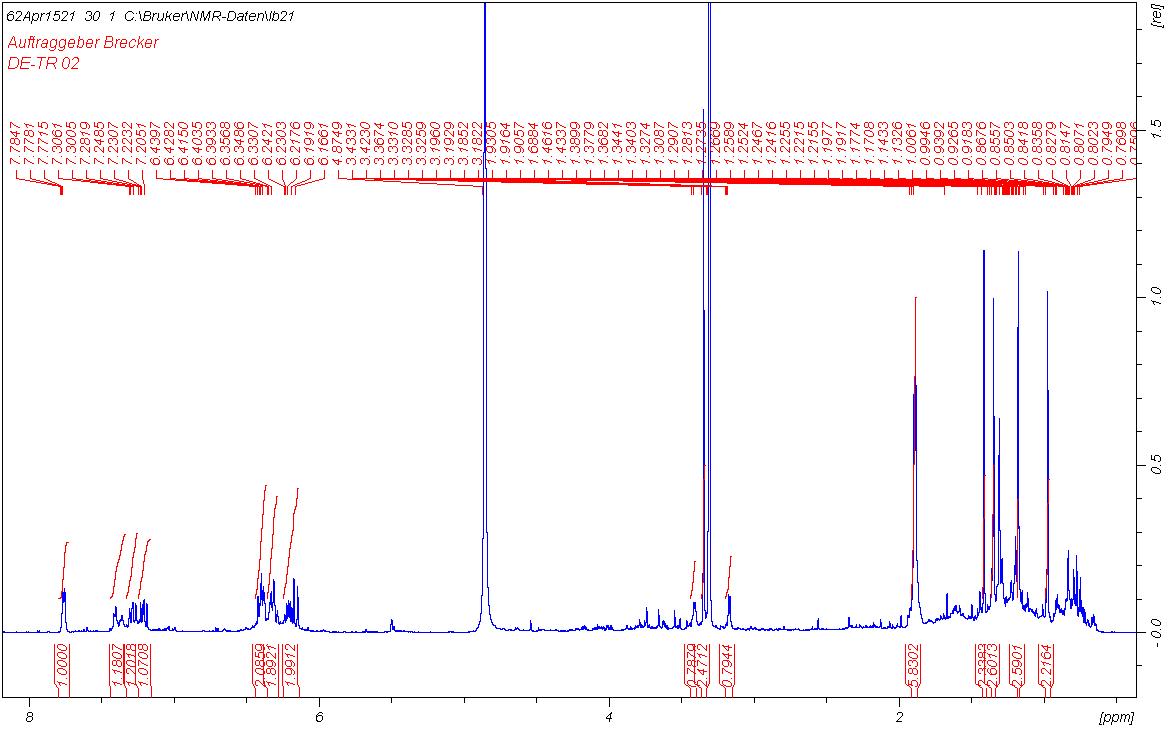


**Supplementary Figure S6.** ^1^H NMR of (21*S*)-bisorbibutenolide in CD_3_OD.

**
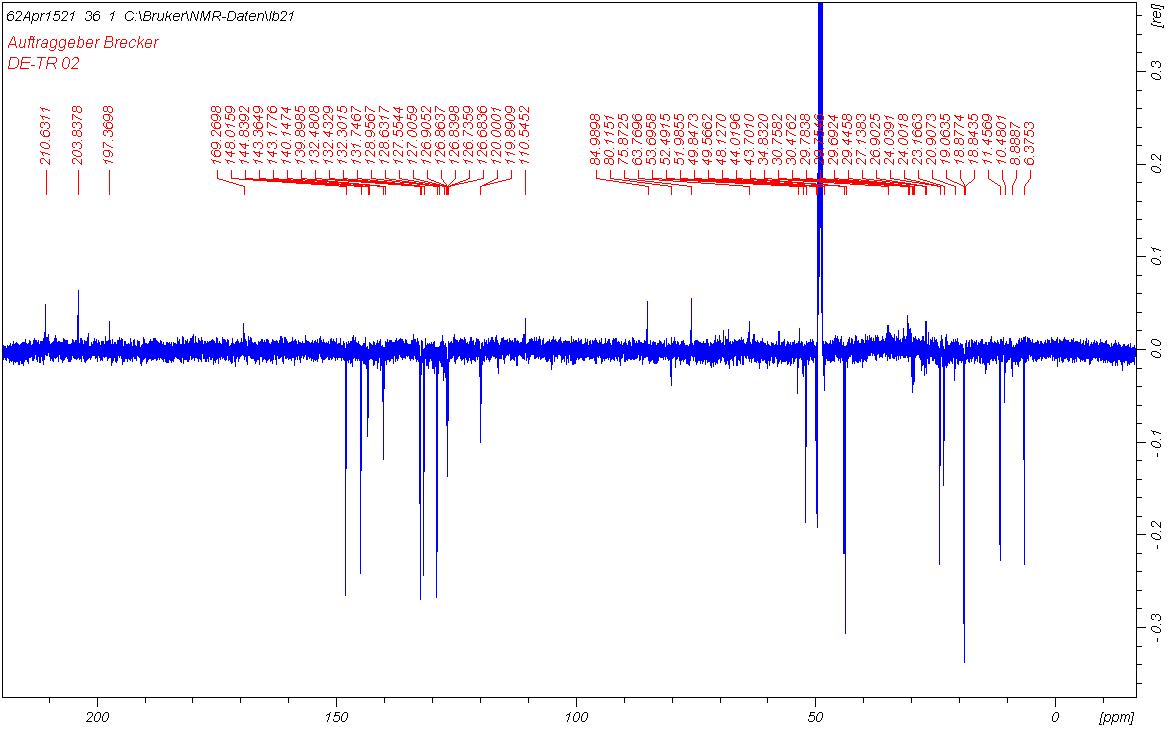
**

**Supplementary Figure S7.** ^13^C NMR [APT] of (21*S*)-bisorbibutenolide l in CD_3_OD.


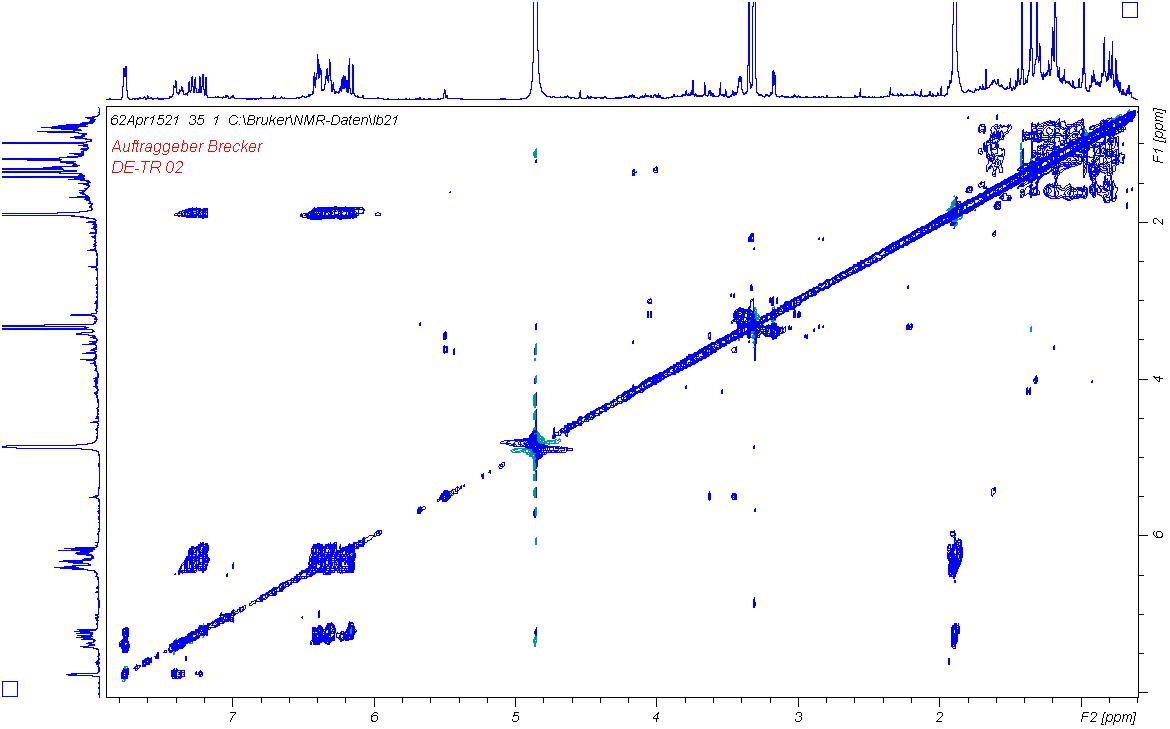


**Supplementary Figure S8.** TOCSY of (21*S*)-bisorbibutenolide in CD_3_OD.


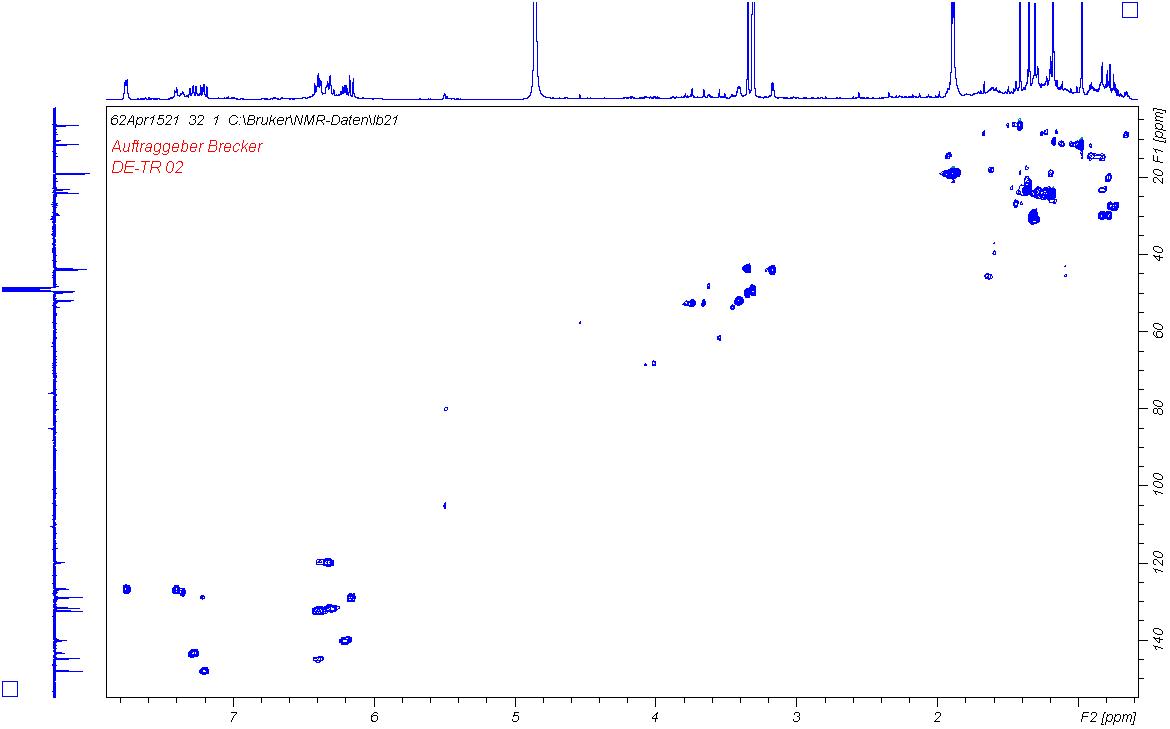


**Supplementary Figure S9.** HSQC of (21*S*)-bisorbibutenolide in CD_3_OD.

**Supplementary Figure S10.** HR-ESI-TOF-MS (negative mode) of (21*S*)-bisorbibutenolide.

**Supplementary Figure S11**. HR-ESI-TOF-MS (positive mode) of (21*S*)-bisorbibutenolide.

**Supplementary Table S1**

| **primer name** | **information** | **Sequence 5' - 3'** | **Protein ID** | **name of target gene** |
| --- | --- | --- | --- | --- |
| 121539_3F | construction of deletion cassette | CTCCTTCAATATCATCTTCTGTCTCCGACGTAGGTGTGGTCAGTGATGG | 121539 | tmk1 |
| 121539_3R | construction of deletion cassette | GCGGATAACAATTTCACACAGGAAACAGCTGTAGCAGTCTCTCTTGTCG | 121539 | tmk1 |
| 121539_5F | construction of deletion cassette | GTAACGCCAGGGTTTTCCCAGTCACGACGTTGATCCCTCTGTGTCAACC | 121539 | tmk1 |
| 121539_5R | construction of deletion cassette | ATCCACTTAACGTTACTGAAATCTCCAACGAACTGAAGAGGAGTTGAGG | 121539 | tmk1 |
| 121539_qF | internal primer | CACGCCCACCATGGAGGACTAC | 121539 | tmk1 |
| 121539_qR | internal primer | GCTCGAGGTACGGGTGCTTGAG | 121539 | tmk1 |
| 82351_3F | construction of deletion cassette | CTCCTTCAATATCATCTTCTGTCTCCGACGTTATCTGAGTGGTGTGTGG | 82351 | tmk2 |
| 82351_3R | construction of deletion cassette | GCGGATAACAATTTCACACAGGAAACAGCCCATGTTCTACGACATCAGC | 82351 | tmk2 |
| 82351_5F | construction of deletion cassette | GTAACGCCAGGGTTTTCCCAGTCACGACGCACCTCTGTATCTCAAGACG | 82351 | tmk2 |
| 82351_5R | construction of deletion cassette | ATCCACTTAACGTTACTGAAATCTCCAACTATGTTGTCTGAGTCCCAGC | 82351 | tmk2 |
| 82351_qF | internal primer | CCAACGAAGAGACTCTCCGCCG | 82351 | tmk2 |
| 82351_qR | internal primer | AAGTTGAACGTCGTGGGGCAGT | 82351 | tmk2 |
| 45018_3F | construction of deletion cassette | CTCCTTCAATATCATCTTCTGTCTCCGACTATAGAGGTCTGGAGGTTGC | 45018 | tmk3 |
| 45018_3R | construction of deletion cassette | GCGGATAACAATTTCACACAGGAAACAGCAGAGAGATACTCCGTCTTCG | 45018 | tmk3 |
| 45018_5F | construction of deletion cassette | GTAACGCCAGGGTTTTCCCAGTCACGACGCTGAAGCTTAGACTTGCTGC | 45018 | tmk3 |
| 45018_5R | construction of deletion cassette | ATCCACTTAACGTTACTGAAATCTCCAACCGCAGGATTATAGCAGTTCC | 45018 | tmk3 |
| 45018_qF | internal primer | GACCCTACTGACGAGCCGGTTG | 45018 | tmk3 |
| 45018_qR | internal primer | CGGGAACTGCTCGTCCATGTTT | 45018 | tmk3 |
| EF1-728F | internal primer | CATCGAGAAGTTCGAGAAGG | 46958 | tef1 |
| TEF1 rev | internal primer | GCCATCCTTGGAGATACCAGC | 46958 | tef1 |
| HRM_SNP3_F | HRM primer | ACAGCATTAGCCCCTTCGTC | 67350 | ham5 |
| HRM_SNP3_R | HRM primer | GCACATTCGCGACAGACTTC | 67350 | ham5 |
| matA1_F | mating type check | TCCTCTCAATGCGTTCATGGC | mat1-1 | mat1-1 |
| matA1_R | mating type check | AGAAGATCATTCTCTGTGTTGGGA | mat1-1 | mat1-1 |
| MATa1-R | mating type check | ATTTGCGCGGCTTGTATTGG | mat1-2 | mat1-2 |
| MATa1-F | mating type check | GCGCACCACGGTATTTCATTG | mat1-2 | mat1-2 |
| RTcbh1F | qPCR primer | ACCGTTGTCACCCAGTTCG | 123989 | cbh1 |
| RTcbh1R | qPCR primer | ATCGTTGAGCTCGTTGCCAG | 123989 | cbh1 |
| RT_VEL_R1 | qPCR primer | GCAGGAACACCAGTCAGGATG | 122284 | vel1 |
| RT_VEL_F1 | qPCR primer | CGAGGAGGGCAAGGACATTAC | 122284 | vel1 |
| SAR RTF1 | qPCR primer/HKG | TGGATCGTCAACTGGTTCTACGA | 61470 | sar |
| SAR RTR1 | qPCR primer/HKG | GCATGTGTAGCAACGTGGTCTTT | 61470 | sar |
| RT_82208_F | qPCR primer | ACTGAAGCAGTATCGGGCAACT | 82208 | pks4 |
| RT_82208_R | qPCR primer | TCTTCGACGTAAAGAGCAGCCA | 82208 | pks4 |
| xyr1RTF | qPCR primer | CTTCCTCCTCCTGCTCATCG | 122208 | xyr1 |
| xyr1RTR | qPCR primer | TCGTGTGCCCTAACAATGGTC | 122208 | xyr1 |
| RT_CRE1 F | qPCR primer | GCAGCACAATACGACTCCG | 120117 | cre1 |
| RT_CRE1 R | qPCR primer | CGGCTAATGATGTCGGTAAG | 120117 | cre1 |
| hpr2F | qPCR primer | TGGCACCACTTCATCAACTTC | 64018 | hpr2 |
| hpr2R | qPCR primer | GGAGTAGGAGGAGGATGTGTTG | 64018 | hpr2 |
